# Atmospheric fallout impact on ^210^Po and ^210^Pb content in wild growing mushrooms

**DOI:** 10.1007/s11356-020-08559-w

**Published:** 2020-04-04

**Authors:** Karolina Szymańska, Dagmara Strumińska-Parulska

**Affiliations:** Laboratory of Toxicology and Radiation Protection, Faculty of Chemistry, University of Gdańsk, Wita Stwosza 63, 80-308 Gdańsk, Poland

**Keywords:** ^210^Po, ^210^Pb, Atmospheric fallout impact, Mushrooms, Bioconcentration, Distribution

## Abstract

The atmospheric fallout impact on ^210^Po and ^210^Pb content in fruitbodies of wild growing mushrooms collected from different environments were investigated. The samples of morphologically different mushroom species, namely bay bolete (*Imleria badia* (Fr.) Vizzini), slippery jack (*Suillus luteus* (L.) Roussel), fairy ring mushroom (*Marasmius oreades* (Bolton) Fr.) and common earthball (*Scleroderma citrinum* Pers.) with their mycelium and soil substrate were collected. Their fruitbodies were separated into cap skin, cap flesh and stem. Also mycelium and soil substrate were collected. The results showed the highest ^210^Po and ^210^Pb activity concentrations were found in *Marasmius oreades* cap skin: 3.20 ± 0.12 and 21.1 ± 0.5 Bq kg^−1^ ww, respectively, which constituted 31.2 and 78.7% of their content in the total fruitbody mass. In the case of open space wild growing mushrooms, their whole caps contain a significantly higher amount of ^210^Po and ^210^Pb when compared to the stem, and their content in the whole cap was determined mainly by concentrations in the cap skin.

## Introduction

Naturally occurring radionuclides give the major contribution to the total effective dose of ionizing radiation of the whole population. Radionuclides are transferred from the site by air emissions, leaching and run-off water, as well as from soils into plants, animals and finally to man (Persson and Holm, 2011; Turtiainen et al., 2013). In case of many elements, mushrooms are known bioaccumulators and at a different level they accumulate stable and radioactive elements (Baeza et al., 2006; Malinowska et al., 2006; Vaaramaa et al., 2009; Falandysz and Borovička, 2013; Szymańska et al. 2018, 2019).(Table 1).

**Table 1:** The average percentage participation of isolated mushroom parts in the whole fruitbody

Species	Stem	Cap flesh	Cap skin
	Percentage participation [% wet mass]		
*Imleria badia*	28.8	43.3	28.0
*Suillus luteus*	18.3	70.4	11.3
*Marasmius oreades*	7.7	80.9	11.4
*Scleroderma citrinum*	2.9	85.1	12.0

Usually, radiological studies have considered mushrooms as a whole fruitbody or divided into cap and stem, and the radionuclides sources and absorption pathways were only a presumption. The previous studies on trace metals showed the proportion of the metal contents originating from the atmosphere depositions seemed to be less important due to the short lifetime of a fruiting body, (usually 10–14 days) and stated metals contents were considerably affected by the age of mycelium and accumulated directly from the soil (Kirchner and Daillant 1998; Thomet et al., 1999; Das, 2005; Kalač and Svoboda 2000; Guillén et al., 2009). But the significant impact of atmospheric fallout was proved in the mosses, the pollutants accumulated in the leaves mostly come from atmospheric deposition, rather than from soil contamination (Długosz-Lisiecka, 2017). The objective of the study is to analyse the ^210^Po and ^210^Pb content in separated into cap skin, cap flesh and stem the fruitbodies of wild growing mushrooms as well as their mycelium and soil substrate collected from different environments, and estimate how their natural growth might affect radionuclides distribution. The field study has been done as the best reflection of the natural conditions impact on the radionuclides uptake—the species selected had different morphology and came from different ecosystems. There were some studies on how fruitbody development can affect the distribution of the radionuclides, but they were done under controlled laboratory conditions (Baeza et al. 2006). The field studies on radiocaesium in *Amanita muscaria* were reported by Falandysz et al. (2019).

Our research focused on isolated parts of wild growing mushrooms collected in their natural environment and addressed the question whether the main source of ^210^Po and ^210^Pb in their fruitbodies is the atmospheric fallout or alternatively a selective bioconcentration from the soil. We also investigated the differences in radionuclides content from different parts of mushrooms.

### Analysed mushrooms characteristic

Bay bolete (*Imleria badia*) grows in coniferous and mixed forests, sometimes just next to stumps, often in moss. The cap is up to 15 cm in diameter with tube hymenophore, and the stipe is thick and 4–9 cm long. *Imleria badia* is predominantly an ectomycorrhizal species that is formed with European spruce (*Picea abies*) and Monterey pine (*Pinus radiata*).

Slippery jack (*Suillus luteus*) widespread, grows in coniferous forests, pioneer species that forms ectomycorrhizal associations with various species of pine. The cap is characteristically slimy in wet conditions, generally 4–10 cm in diameter with tube hymenophore, and the stipe is 5–10 cm tall. Fairy ring mushroom (*Marasmius oreades*) grows in the open space above the grasses. The cap is faintly lined and 1–5 cm in diameter with lamellae hymenophore, and the stem is thin, long and fibrous and grows up to about 7 cm. Saprophytic. Common earthball (*Scleroderma citrinum*) widespread and popular, grows on the ground in sandy and acidic soils, in the woods and also outside forests. Mycorrhizal without any strict preferences. The *Scleroderma citrinum* fruitbodies do not have an open cap containing spore-bearing gills (spores are produced internally forming the gleba). The fruitbody is irregularly spherical or bulbous, up to 10 cm in diameter with glebal hymenium. It has no typical stem, but a short pseudostem (subgleba) (Gumińska and Wojewoda, 1968).

## Materials and methods

The samples of four morphologically different mushroom species, namely bay bolete (*Imleria badia* (Fr.) Vizzini), slippery jack (*Suillus luteus* (L.) Roussel), fairy ring mushroom (*Marasmius oreades* (Bolton) Fr.) and common earthball (*Scleroderma citrinum* Pers.) with their mycelium and soil substrate taken from up to 10 cm depth, were collected. All analysed mushrooms, different in their morphology as well as living in different environmental conditions were chosen intentionally. *Imleria badia* with mycelium and its soil substrate was collected from a forested, sheltered place close to Włocławek (central Poland; coordinates 52.637226 and 19.065876); while *Suillus luteus*, *Marasmius oreades* and *Scleroderma citrinum* with their mycelium and soil substrate were taken from grasslands in Gdańsk (northern Poland; respective coordinates 54.337586 and 18.552243; 54.394300 and 18.575254 as well as 54.327620 and 18.600475). The fruitbodies of *Suillus luteus* and *Scleroderma citrinum* grew uncovered in the grass, in the vicinity of the tree, while the fruitbodies of *Marasmius oreades* grew in a completely open space, directly exposed to atmospheric fallout.

### Samples preparation

In the laboratory, all samples were cleaned from all visible impurities as plant and animal organisms. Three analytical samples were isolated from the collected primary samples and each separated mushroom analytical sample contained 7–10 mushroom fruitbodies. From fresh mushroom fruitbodies, using a ceramic knife, the skin of the cap, cap flesh and stem were separated. In the case of *Scleroderma citrinum* we were able to achieve mushroom skin while its gleba was treated as cap flesh (pileus that supports hymenium) and the subgleba as the stem. The visible mycelium from the soil substrate was separated manually. The isolated parts were weighed individually and their participation in the mass of the fruitbody was determined (Table 1).

### Radiochemical analysis

Fresh mushroom samples were used during the radiochemical analysis. The primary samples collected at sampling sites were divided into three analytical samples and their masses, depending on the species, were 7–170 g of separated mushroom parts, 4.5–8 g of mycelium and 2.5–6 g of soil substrate. Each analytical sample we obtained was enriched with 9 mBq of ^209^Po as a yield tracer. All materials were digested using conc. (65%) HNO_3_ and heated until acid has evaporated. The dried residue was dissolved in 0.5 M HCl and about 0.2 g of ascorbic acid was added. The polonium measurement discs were prepared by its autodeposition on pure 100% silver in 90 °C and the activities of ^209^Po and ^210^Po were measured using an alpha spectrometer (Strumińska-Parulska, 2015; Strumińska-Parulska and Olszewski, 2018). The ^210^Pb determination method was based on its indirect measurement via its daughter ^210^Po activity measurement. After the first polonium deposition, evaporated and dry samples were stored for 10–12 months to achieve a sufficient ^210^Po ingrowth from ^210^Pb. Next, each sample was treated with 9 mBq of ^209^Po tracer again and digested like before. The deposition of ^210^Po on the silver disc was repeated and the activities of ingrowing ^210^Po were measured in the alpha spectrometer. The ^210^Pb activity was calculated using the simplified form of the Bateman equation (Skwarzec, 1997).

The chemical analysis efficiency of ^210^Po and ^210^Pb determination ranged 90–98%; the activity concentrations of ^210^Po and ^210^Pb in analysed mushrooms, mycelium and soil samples were calculated on the sampling time; and the results were given with standard deviation (SD) calculated for 95% confidence intervals. In both cases, the single measurement took 1–3 days. The minimum detectable activity (MDA) was 0.10 mBq. Due to the small amount of samples, all statistical procedures were based on non-parametric tests, mainly the U test (Mann-Whitney) and H test (Kruskal-Wallis).

### Bioconcentration factor (BCF), distribution ratio (DR) and normalised partition factor (PF)

We were also interested in chemical elements uptake and their bioconcentration from the soil, water or air, as well as distribution (fate) in a fruitbody. Therefore, in the case of analysed fungi samples, we calculated the values of the bioconcentration factor (BCF) and the discrimination ratio (DR). The soil-mushroom radionuclides bioconcentration level was assessed by BCF (Gadd, 2007; Strumińska-Parulska et al., 2016). The radionuclides distribution into the caps and stems of analysed mushroom fruitbodies was assessed using discrimination ratio—a value of DR > 1 shows that is preferred by species distribution of a radionuclide into parts of the fruitbodies (Baeza et al., 2006). The value of DR > 1 might also indicate the atmospheric fallout impact on radionuclides’ presence in the cap. In the case of mushrooms, we cannot use well-known translocation factor (designed for plants), due to the hyphal structure of the fruitbodies (Trotta et al., 2006; Yu et al., 2012).

The other good coefficient useful in describing the distribution of the radionuclides in analysed organisms can be the normalised partition factor (PF). The PF for analysed mushroom parts (cap skin, cap flesh, stem) is defined as a ratio of ^210^Po and ^210^Pb percentage contribution in the analysed part to the percentage mass contribution of this element in the whole organism. The PF values in analysed mushroom pieces reflected the radionuclide distribution in the mushroom organisms; PF > 1 indicates effective radionuclide accumulation (or increased content) in organ or tissue of the analysed organism (Strumińska-Parulska et al. 2011).

## Results and discussion

### ^210^Po and ^210^Pb activity concentrations

In order to explain the sources of bioconcentration of ^210^Po and ^210^Pb, and thus to examine the influence of atmospheric fallout on their content in particular parts of fruiting bodies, wild-growing forest and meadow mushroom samples were collected and first ^210^Po and ^210^Pb activity concentrations have been determined. The first information we get looking at the results is the values of ^210^Po and ^210^Pb activity concentrations in *Marasmius oreades* and *Scleroderma citrinum* are the highest in their cap skin, while in *Imleria badia* spiecies in its stem.

When analysing the average values of ^210^Po concentrations in mushroom fruitbodies, the highest ^210^Po activity has been determined in *Marasmius oreades* cap skin (3.20 ± 0.12 Bq kg^−1^ ww), while the lowest in *Scleroderma citrinum* cap flesh (0.18 ± 0.01 Bq kg^−1^ ww). The data has shown higher ^210^Po activity concentrations in stems of *Imleria badia* and *Suillus luteus* (1.78 ± 0.07 and 1.17 ± 0.07 Bq kg^−1^ ww, respectively) (Table 2). The fruitbodies of *Scleroderma citrinum* and *Suillus luteus* were collected near the tree (but not covered), so their exposures were not limited significantly. The most limited impact of atmospheric fallout was in the case of *Imleria badia* samples that were collected in the forest. *Marasmius oreades* and *Scleroderma citrinum* are quite specific types of mushrooms. *Marasmius oreades* grows in the open space above the grasses and his cap far exceeds the vegetation occurring in the surroundings and is relatively large when compared to the stem, hence the exposure to weather conditions is the greatest. The *Scleroderma citrinum* fruitbodies do not have the typical open cap, but spherical gleba, and have short pseudostem (subgleba) (Gumińska and Wojewoda, 1968).

**Table 2:** The average values of ^210^Po and ^210^Pb activity concentrations in analysed samples and their percent contribution in the whole fruitbody content

Sample (*n*; *i*)^1^	Activity concentration (Bq kg^−1^ wet ± SD) (Percent contribution in fruitbody (% ± SD))					
	Whole fruitbody^2^	Cap skin	Cap flesh	Stem	Mycelium	Soil
^210^Po						
*Imleria badia* (3; 7–10)	1.01 ± 0.05 (100.0)	0.89 ± 0.06 (24.7 ± 1.7)	0.59 ± 0.03 (25 ± 1.4)	1.78 ± 0.07 (50.3 ± 1.8)	91.7 ± 2.0	172 ± 4
*Suillus luteus* (3; 7–10)	0.74 ± 0.03 (100.0)	0.79 ± 0.05 (11.9 ± 0.7)	0.63 ± 0.02 (59.3 ± 1.7)	1.17 ± 0.07 (28.8 ± 1.8)	48.3 ± 1.7	39.9 ± 1.2
*Marasmius oreades* (1; 8)	1.17 ± 0.05 (100.0)	3.20 ± 0.12 (31.2 ± 1.2)	0.93 ± 0.04 (64.3 ± 2.4)	0.69 ± 0.05 (4.5 ± 0.3)	17.5 ± 0.4	35.2 ± 0.7
*Scleroderma citrinum* (3; 7–8)	0.23 ± 0.01 (100.0)	0.61 ± 0.04 (31.5 ± 2.0)	0.18 ± 0.01 (64.3 ± 5.1)	0.34 ± 0.02 (4.2 ± 0.2)	22.2 ± 1.7	29.1 ± 0.9
^210^Pb						
*Imleria badia* (3; 7–10)	0.88 ± 0.04 (100.0)	0.68 ± 0.04 (21.6 ± 1.2)	0.36 ± 0.02 (17.9 ± 0.9)	1.84 ± 0.07 (60.5 ± 2.4)	74.8 ± 1.3	142 ± 2
*Suillus luteus* (3; 7–10)	0.17 ± 0.01 (100.0)	0.48 ± 0.03 (32.4 ± 1.8)	0.08 ± 0.01 (34.0 ± 1.9)	0.31 ± 0.01 (33.6 ± 1.6)	29.7 ± 1.4	37.0 ± 1.5
*Marasmius oreades* (1; 8)	3.06 ± 0.09 (100.0)	21.1 ± 0.5 (78.7 ± 1.7)	0.72 ± 0.04 (19.1 ± 1.2)	0.86 ± 0.06 (2.2 ± 0.1)	16.1 ± 0.7	47.0 ± 2.4
*Scleroderma citrinum* (3; 7–8)	0.18 ± 0.01 (100.0)	0.80 ± 0.06 (63.3 ± 4.9)	0.11 ± 0.01 (16.1 ± 1.2)	0.47 ± 0.04 (20.6 ± 1.6)	15.2 ± 1.8	36.8 ± 2.4

^1^(*n*; *i*) number of samples; number of individuals in each sample

^2^value calculated on the basis of skin, cap and stem contribution in the whole fruitbody

The research on ^210^Pb content in the fruiting bodies of the analysed mushrooms has indicated its highest concentration also in *Marasmius oreades* cap skin (21.1 ± 0.5 Bq kg^−1^ ww) and the lowest in *Suillus luteus* cap flesh (0.08 ± 0.01 Bq kg^−1^ ww). In the case of *Marasmius oreades*, *Suillus luteus* and *Scleroderma citrinum*, the ^210^Pb concentrations in stems have been lower when compared with their skin. However, in the case of *Imleria badia*, the concentration of ^210^Pb has been significantly higher in the stem (Table 2). It means that the ^210^Pb accumulation from the soil substrate has been the leading process in *Imleria badia*. It might also show a leading influence of ^210^Pb accumulation from the soil due to limited exposure to atmospheric fallout and potential ^210^Pb adsorption due to covered, forested area.

### ^210^Po and ^210^Pb bioconcentration factors (BCFs), distribution ratios (DRs) and normalised partition factor (PF)

In order to reveal the degree of accumulation of ^210^Po and ^210^Pb from the soil substrate by the mushroom fruiting bodies, the bioconcentration factor (BCF) was used, whereas the calculated distribution ratio (DR) expressed the potential ^210^Po and ^210^Pb migration inside the fruiting body or atmospheric fallout impact (Table 3). But we decided to check the differences in more detail and compared different, previously separated, parts of the mushrooms. The respective values of concentrations ratio are given as the exact numerators and denominators (Table 3). The normalised partition factor (PF) for analysed mushroom parts (cap skin, cap flesh, stem) was used to reflect effective accumulation of ^210^Po and ^210^Pb in analysed mushroom parts (Table 4). It should be mentioned here that the distribution of a chemical element between cap and stem may undergo a change as fruitbody grows up—its content can be not only as the effect of increased selective bioconcentration, but also as its dilution. It has been previously observed for ^137^Cs and ^40^K in wild growing *Amanita muscaria*, where a decrease of ^137^Cs but not of the essential ^40^K in mature fruitbodies took place (Falandysz et al., 2019).

**Table 3:** The average values of ^210^Po and ^210^Pb bioconcentration factor (BCF) and distribution ratio (DR) in analysed samples

Sample	Bioconcentration factor (BCF)				Distribution ratio (DR)		
	Respective concentrations ratio						
	Mycelium/Soil	Stem/Soil	Stem/Mycelium	^*^Whole fruitbody/Soil	Cap/Stem	Skin/Cap	^*^Skin + Cap/Stem
^210^Po							
*Imleria badia*	0.53	0.01	0.02	0.01	0.33	1.52	0.40
*Suillus luteus*	1.21	0.03	0.02	0.01	0.54	1.26	0.56
*Marasmius oreades*	0.50	0.02	0.04	0.02	1.35	3.45	1.76
*Scleroderma citrinum*	0.83	0.02	0.02	0.01	0.52	3.47	0.68
^210^Pb							
*Imleria badia*	0.53	0.01	0.02	0.001	0.20	1.89	0.26
*Suillus luteus*	0.80	0.01	0.01	0.001	0.26	6.00	0.42
*Marasmius oreades*	0.34	0.02	0.05	0.06	0.84	29.2	3.76
*Scleroderma citrinum*	0.41	0.01	0.03	0.001	0.23	7.27	0.32

*Value calculated on the basis of skin, cap and stem content

**Table 4:** The normalised partition factor (PF) for ^210^Po and ^210^Pb in analysed parts of mushrooms

Sample	Normalized partition factor		
	Skin	Cap	Stem
^210^Po			
*Imleria badia*	0.88	0.58	1.75
*Suillus luteus*	1.05	0.84	1.57
*Marasmius oreades*	2.74	0.79	0.58
*Scleroderma citrinum*	2.63	0.76	1.45
^210^Pb			
*Imleria badia*	0.77	0.41	2.10
*Suillus luteus*	2.87	0.25	0.48
*Marasmius oreades*	6.90	0.22	0.29
*Scleroderma citrinum*	5.28	0.19	7.10

The ^210^Po and ^210^Pb bioconcentration factor (BCF) values range from 0.01 to 1.21 for ^210^Po and from 0.001 to 0.80 for ^210^Pb. The highest value of BCF, and higher than 1, have been calculated for *Suillus luteus* mycelium to soil ratio in the case of ^210^Po (Table 3) what indicated low but effective accumulation of these radionuclides. In the case of other samples, the accumulation has been very low and the study confirmed the low level of ^210^Po and ^210^Pb accumulation—despite their significant content in the soil and mycelium, there is a relatively low concentration in the stem. Also much higher ^210^Po and ^210^Pb activity concentrations in soil collected in Włocławek were measured, but they have not influenced on their higher bioconcentration by *Imleria badia* (Table 3). Although both ^210^Po and ^210^Pb were present in the analysed mushrooms, they were poorly accumulated, and it was previously observed and reported (Kirchner and Daillant 1998; Guillén et al., 2009; Brzostowski et al., 2011; Jarzyńska and Falandysz, 2012; Gwynn et al., 2013; Turtiainen et al., 2013; Siric et al., 2016). The results obtained in the study confirm earlier reports of a significantly lower ^210^Pb accumulation capacity compared to the ^210^Po (Kalač and Svoboda, 2000). As a result, the distribution and accumulation of more reactive ^210^Po is slightly more effective than ^210^Pb, but both processes (distribution and accumulation from the soil by the mycelium) are low, and ^210^Po as well as ^210^Pb are not selectively bioaccumulated. We suppose that the content of ^210^Po and ^210^Pb in the fruitbody is a result of its dilution when mycelium grows and forms the sporocarp.

The ^210^Po and ^210^Pb distribution ratio (DR) values range from 0.33 to 3.47 for ^210^Po and from 0.20 to 29.2 for ^210^Pb. The highest values of DR, and higher than 1, have been calculated for every mushroom cap skin to cap flesh ratio (Table 3). In the case of open space wild growing mushrooms, these values are higher when compared to *Imleria badia* from the forest, especially considering ^210^Pb (U test *p* value is 0.04 for ^210^Pb DRs). The DR values of “cap flesh to stem” as well as “the whole cap to stem” show there is no effective accumulation of ^210^Po and ^210^Pb from stem to a cap. In the case of *Marasmius oreades*, DR values imply some effective transport of ^210^Po and ^210^Pb to cap flesh that were adsorbed on the skin, although further research is needed to clarify the issue. Still, the obtained DR values allow us to state the main source of ^210^Po and ^210^Pb in mushroom caps is the atmospheric fallout or other substances present in the mushroom environment, such as soil or dust particles carried by the wind. But the main source of ^210^Po and ^210^Pb contained in the cap has not been the bioaccumulation process from the soil.

On the basis of obtained normalised partition factor (PF) values (Table 4), we could notice that the cap skin samples contained the highest amount of ^210^Po and ^210^Pb when compared to their share of the whole mushroom. However, the PF values for cap skin and the stem indicate that there is no statistically significant differences in ^210^Po accumulation (U test *p* = 0.15), while in the case of ^210^Pb the skin of the cap contains more ^210^Pb (U-test *p* = 0.04). On the basis of obtained results, we suppose that about 79% and 63% of ^210^Pb in the cap skin of *Marasmius oreades* and *Scleroderma citrinum,* respectively, is adsorbed and comes from the atmosphere (the environment). The rest of ^210^Pb has been built in the mushroom structure due to accumulation processes. We can conclude that the presence of ^210^Po and ^210^Pb in the stem is a result of effective bioaccumulation, ^210^Po and ^210^Pb amount in the cap reflects dilution process, while their content in the cap skin is strictly connected to the atmospheric fallout.

### ^210^Po and ^210^Pb dominant sources

**Fig. 1:**
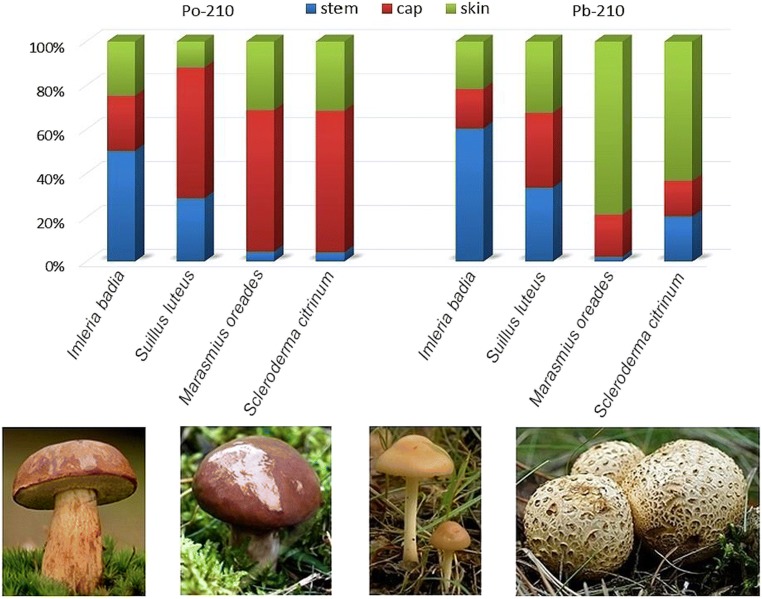
Isolated mushroom parts share in ^210^Po and ^210^Pb distribution

The obtained results indicate that the total content of ^210^Po and ^210^Pb in open space wild growing mushrooms (*Suillus luteus*, *Marasmius oreades* and *Scleroderma citrinum*) is determined to a large extent by their amount in the whole cap (cap flesh + skin) (Table 2). In the case of *Suillus luteus*, *Marasmius oreades* and *Scleroderma citrinum*, ^210^Po originating from the soil is in a smaller share. In the case of *Marasmius oreades* and *Scleroderma citrinum*, atmospheric fallout is the main source of ^210^Po—its content in the skin is 31.2 and 31.5%, respectively, while the cap skin constitutes 11.4 and 12.0% of the total weight of the fruiting body, respectively (Table 2; Fig. 1). Opposite, in *Imleria badia* samples collected from the forest ecosystem, the highest ^210^Po content has been found in the stem (50.3%) while the smallest in the skin cap (24.7%). Considering the fact that the *Imleria badia* stem constitutes 28.8% of the total fruiting body fresh weight, 50.3% of the total content of ^210^Po a significant value (Table 2; Fig. 1). The results indicate that due to the habitat sheltered by, the dominant source of ^210^Po is its accumulation from the soil, and the atmospheric fallout has a much smaller impact.

In the case of ^210^Pb, *Imleria badia* contains its highest amount in the stem as well (60.5%) and the soil is the dominant source of ^210^Pb (Table 2; Fig. 1). In the fruiting body of *Suillus luteus*, ^210^Pb is distributed uniformly (32.4–34.0%), but its stem constitutes 18.3%, while the skin 11.3% of the fruiting body total weight. It might be concluded that the ^210^Pb accumulation from soil through the mycelium, as well as the atmospheric fallout, are sources with a similar share. In the case of *Marasmius oreades* and *Scleroderma citrinum*, the largest share in ^210^Pb content in the fruiting body is cap skin (78.7 and 63.3%, respectively), especially considering that the cap skin of these species constitutes 11.4 and 12.0% of the total fruiting bodies weight, respectively (Table 2; Fig. 1). This suggests that the atmospheric fallout can be an important source of ^210^Pb. Opposite, radiolead coming from the soil, accumulated by the mycelium and transported through the stem, may have much smaller contribution to the entire accumulation of the radionuclide. In the case of *Scleroderma citrinum*, spherical sporocarps growing close to the earth’s surface may be of special importance—^210^Pb might come not only from atmospheric fallout but also in the form of soil particles carried by the wind and adsorbed on the closed fruitbody.

Previous studies suggested that the accumulation from the soil was the main source of ^210^Po and ^210^Pb (Kirchner and Daillant 1998; Kalač and Svoboda 2000; Guillén et al. 2009). Our results indicated a possible atmospheric origin of ^210^Po and ^210^Pb in mushroom fruiting bodies despite their rapid growth. Opposed to a soil-based origin, in favourable environmental conditions (open space, poor plant cover or forest litter) the atmospheric fallout can be very important and might be the dominant source of ^210^Po and ^210^Pb.

## Conclusions

Although both ^210^Po and ^210^Pb are present in the analysed mushrooms, they are poorly accumulated—despite their significant content in the soil and mycelium, there is a relatively low concentration in the stem. Also, both ^210^Po and ^210^Pb are not selectively bioaccumulated, and the content of ^210^Po and ^210^Pb in the fruitbody is a result of its dilution when mycelium grows and forms the sporocarp.

The study shows that the atmospheric fallout can play an important role in the uptake and distribution of ^210^Po and ^210^Pb in analysed mushroom fruitbodies. Our results indicated a possible atmospheric origin of ^210^Po and ^210^Pb in mushroom fruiting bodies despite their rapid growth, especially in open space wild growing mushrooms. We could conclude that the presence of ^210^Po and ^210^Pb in the stem might be a result of effective bioaccumulation, ^210^Po and ^210^Pb content in the cap reflected dilution process, while their content in the cap skin was strictly connected to the atmospheric fallout. Although it was thought that metal contents originating from the atmosphere depositions seemed to be less important, the research has indicated the atmospheric fallout could be an important source of ^210^Po and ^210^Pb in wild growing mushrooms.
